# Responsiveness to societal needs in postgraduate medical education: the role of accreditation

**DOI:** 10.1186/s12909-020-02125-1

**Published:** 2020-09-28

**Authors:** Ingrid Philibert, Danielle Blouin

**Affiliations:** 1 Department of Medical Education, Frank H. Netter MD School of Medicine at Quinnipiac University, North Haven, CT USA; 2 Faculty of Health Sciences (Department of Emergency Medicine) and Faculty of Education, Queen’s University, Kingston, Ontario Canada

**Keywords:** Accreditation, Diversity, Equity, Postgraduate medical education, Societal accountability

## Abstract

**Background:**

Social accountability in medical education has been defined as an obligation to direct education, research, and service activities toward the most important health concerns of communities, regions, and nations. Drawing from the results of a summit of international experts on postgraduate medical education and accreditation, we highlight the importance of local contexts in meeting societal aims and present different approaches to ensuring societal input into medical education systems around the globe.

**Main text:**

We describe four priorities for social responsiveness that postgraduate medical education needs to address in local and regional contexts: (1) optimizing the size, specialty mix, and geographic distribution of the physician workforce; (2) ensuring graduates’ competence in meeting societal goals for health care, population health, and sustainability; (3) promoting a diverse physician workforce and equitable access to graduate medical education; and (4) ensuring a safe and supportive learning environment that promotes the professional development of physicians along with safe and effective patient care in settings where trainees participate in care. We relate these priorities to the values proposed by the World Health Organization for social accountability*: relevance, quality, cost-effectiveness, and equity;* discuss accreditation as a lever for change; and describe existing and evolving efforts to make postgraduate medical education socially responsive.

**Conclusion:**

Achieving social responsiveness in a competency-based postgraduate medical education system requires accrediting organizations to ensure that learning emphasizes relevant competencies in postgraduate curricula and educational experiences, and that graduates possess desired attributes. At the same time, institutions sponsoring graduate medical education need to provide safe and effective patient care, along with a supportive learning and working environment.

## Background

The medical education enterprise is accountable to the citizens who will be served by the physician workforce it produces [1]. An appropriately sized, skilled, and distributed physician workforce is critical to the health of any nation. The composition and skill set of this workforce is determined in significant part during the postgraduate phase of medical education. Three decades ago, the World Federation for Medical Education (WFME) declared that the aim of medical education is to produce physicians who will promote the health of all people, and noted that this aim was not being met despite progress in medical science [2]. In 1995, the World Health Organization (WHO) defined the social accountability of medical schools as “the obligation to direct their education, research and service activities towards addressing the priority health concerns of the community, region, and/or nation they serve” [3]. Medical education and accreditation experts discussed the importance of social accountability at the 2013 World Summit on Outcomes-Based Accreditation.^1^ Participants at the summit discussed the role of accreditation in a wide range of domains, focusing on the accreditation systems in different nations, including the United States, Canada, and the Netherlands. In this article, we propose “social responsiveness” as a more actionable term for postgraduate medical education, given its relatively decentralized operation in most nations’ health care systems. Social responsiveness has been defined as the “engagement in a course of actions responding to social needs” [4]. Inherent in the WFME and WHO definitions of social accountability are expectations for the competencies of the physician workforce that the medical education system produces. Yet the general nature of the definitions makes it challenging to translate them into actionable recommendations for postgraduate medical education programs and institutions. The term “responsiveness” is congruent with the WHO expectation that health care priorities should be collectively identified by stakeholders, including governments, health care organizations, educators, professionals, and members of the public [3].

## Main text

### Social responsiveness in postgraduate medical education

In contrast to earlier commentators who have discussed social accountability expectations for medical schools, we focus on physician education *after* medical school and describe four priorities for social responsiveness in the postgraduate medical education system. The first pertains to ensuring that the size, specialty mix, and geographic distribution of the physician workforce is adequate to meet patient and population health needs [5, 6]. The second priority pertains to the skill set necessary to enable the profession to meet societal goals for access, population health, and the stewardship of resources [7] while addressing health disparities [8] and the social determinants of health [9] in increasingly diverse populations. The third priority relates to enhancing the diversity and inclusiveness of the learning environment; this includes increasing the representation of racial, ethnic, and other minorities in the physician workforce and promoting an inclusive and supportive environment for all trainees. The fourth priority pertains to ensuring a safe, respectful, and supportive learning and working environment that facilitates the professional development of physicians while providing safe and effective care in settings where trainees learn and participate in care. Detail on the four priorities is shown in Tables 1, 2, 3 and 4. Three of the priorities relate to the future physician workforce, and one to the learning environment that provides the context for the professional socialization of the future physician workforce. These priorities complement Boelen’s social obligation scale and facilitate its operationalization [4].

**Table 1:** Priority 1: Appropriate size, specialty mix, and geographic composition of the physician workforce

Interventions	Benefits to the health care system (relevant social accountability values)	Strategies to effect change
Ensure appropriate size, specialty mix, and skill set of the physician workforce	Improved access to care for all individuals; improved population health (relevance, equity) [3]	**System:** Top-down government control of specialty choice; national/state/provincial funding incentives for training generalist^a^ physicians **Accreditation:** Standards that require curricula and learning experiences related to improving population health **Institution and program:** Institutions and programs with a mission to train generalist physicians **Individual:** Educational debt forgiveness or incentive payments for individuals selecting generalist specialties
Optimize geographic distribution of physicians	Access to care and improved population health for rural and underserved inner-city populations (relevance, equity) [3]	**System:** Top-down governmental control of health care; national/state/provincial funding incentives for training physicians for rural and underserved locations **Institution and program:** Institutions and programs with a mission to prepare physicians for practice in rural and underserved areas **Individual:** Educational debt forgiveness or incentive payments for individuals practising in rural or underserved areas
Reduce “brain drain” through international medical migration	Enhanced retention of physician workforce in nations with physician shortages; increased international equity and fairness (relevance, equity) [3]	**Global systems:** WHO Global Code of Practice on the International Recruitment of Health Personnel [10]

^a^Generalist specialties include primary care specialties and other general specialties in short supply, such as psychiatry and surgery

**Table 2:** Priority 2: Physician competencies for meeting societal goals for health care and population health at a sustainable cost

Interventions	Benefits to the health care system (relevant social accountability values)	Strategies to effect change
Ensure the quality and safety of health care	Reduced medical errors, improved quality, including better patient experience of care (quality, cost-effectiveness) [3]	**Accreditation:** Standards that promote quality and safety in curricula, improvement projects, and role modelling by teaching faculty; approaches to promote quality and safety improvement in the learning environment **Institution and program:** Institutional- and program-level quality and safety curricula, experiences, and improvement projects
Address health disparities and economic, educational, and **social** conditions that influence health status	Increased access to care; improved health care equity (relevance, equity) [3]	**Accreditation:** Standards requiring programs to set and meet aims relevant to the needs of the communities they serve; approaches to highlight and address health disparities and promote health equity [11] **Institution and program:** Initiatives to teach, role- model, and assess physician competencies important to patient advocacy [12, 13]; measures to address health disparities [14, 15], social determinants of health [16], and system-level factors that create barriers to health and health care for some members of society
Provide resource-conscious care	Stewardship of finite health care resources (cost-effectiveness) [3]	**System:** National initiatives such as the US Choosing Wisely campaign [17] **Accreditation:** Accreditation standards highlighting resource-consciousness in teaching and trainee assessment

**Table 3:** Priority 3: Diversity of physician workforce, and diversity and inclusiveness in the learning and working environment

Interventions	Benefits to the health care system (relevant social accountability values)	Strategies to effect change
Promote equity in access to medical education for all individuals regardless of gender or racial, ethnic, or other minority status	Increased fairness; enhanced diversity; a physician workforce that is more representative of the diversity of patients (relevance, equity) [3]	**System:** Affirmative action [18] (despite ongoing challenges) [19] **Accreditation:** Accreditation requirements that promote diversity and inclusion [20]; standards for programs and institutions to focus on the ongoing, systematic recruitment and retention of a diverse workforce, including trainees, faculty, senior leaders, and other members of the academic community **Institution and program:** Institutions with a mission to educate members of racial or ethnic minorities; holistic admissions approaches that promote diversity and inclusion [21]
Promote cultural curiosity, sensitivity, and humility	Improved experience of care; promotion of patient engagement and adherence to care (relevance, quality, equity) [3]	**Accreditation:** Accreditation efforts to highlight cultural sensitivity and cultural competence in curricula, educational programming, faculty role modelling, and trainee assessment [22] **Institution and program:** Institutional- and program-level efforts to teach, role-model, and assess cultural competency and sensitivity
Promote diversity and inclusion with respect to race, ethnicity, religion, gender, and sexual orientation in the medical workforce	Promotion of an inclusive, supportive learning environment, and of cultural sensitivity and humility in the learning environment and in graduates’ future practice	**Accreditation:** Accreditation standards requiring that programs and their institutions focus on the ongoing, systematic recruitment and retention of a diverse workforce, including trainees, faculty, senior leaders, and other members of the academic community. **Institution and program:** Institutional- and program-level efforts to teach, role-model, and assess inclusiveness in the learning and working environment; faculty development focused on the advancement of minorities [23]

**Table 4:** Priority 4: A safe, supportive learning environment that facilitates the co-production of professional development for physicians and safe and effective patient care

Ensure a respectful, supportive, and caring environment for trainees, faculty, and staff	Promotion of the professional and personal development of learners; improved work environment for learners/faculty/staff (quality, equity) [3]	**Accreditation:** Accreditation standards against harassment of trainees; standards for trainees and others to raise concerns with their learning and working environment **Institution and program:** Institutional- and program-level efforts to promote respect and caring in the learning and working environment
Promote the safety and effectiveness of patient care in teaching settings	Appropriate supervision and oversight of care; improved experience of care for patients (quality, equity) [3]	**Accreditation:** Accreditation standards limiting trainee work hours; standards to educate trainees and faculty and fatigue management; standards for appropriate supervision and faculty oversight of care in teaching settings, and equity of care regardless of patients’ payer status **Institution and program:** Institutional- and program-level efforts to promote trainee alertness and fitness to provide care; programs to provide appropriate supervision and faculty oversight of care
Enhance the well-being of trainees, faculty, and other participants in the learning environment	Reduced burnout, distress, and depression in residents, faculty, and other professionals; enhanced work-life balance (quality, equity) [3]	**Accreditation:** Accreditation standards for trainee and faculty wellness programs; trainee access to care programs for physical and mental care and counselling **Institution and program:** Institutional programs to provide care, counselling, and suicide prevention for trainees and faculty in distress [24]; programs to address the consequences of patient death, medical error, or other adverse events for residents and faculty [25]

### Approaches to meeting social responsiveness aims

In Tables 1, 2, 3 and 4, we summarize interventions at the level of the system (government entities and accreditors), the institution and program, and the individual to address the four priorities. This highlights accreditation as a lever for social accountability in many domains. We relate the social responsiveness priorities we identified to four values for social accountability discussed in a 1995 WHO document: (1) relevance (the degree to which the aims of medical education are congruent with the needs and aims of the communities it serves); (2) quality (the degree to which medical education contributes to high-quality, effective care, both in the settings where trainees participate in care and in graduates’ future practice); (3) cost-effectiveness (the extent to which medical education contributes to cost-effective care); and (4) equity (making health care available to all, and making medical education accessible to women and members of racial, ethnic, and other minorities) [3].

The four social responsiveness priorities shown in Tables 1, 2, 3 and 4 are not new, and many of the aspects summarized there are congruent with a consensus document on the social responsibility of medical schools [26]. Yet, despite a general appreciation and considerable agreement on dimensions of social accountability, few studies have broadly addressed social responsiveness in postgraduate medical education. Work to date has focused on efforts to address a single aspect, such as optimizing the size and distribution of the physician workforce [27], enhanced funding for primary care and rural training programs [28], and service learning projects to expose learners to settings that provide care for underserved patients [29]. Efforts to enhance the postgraduate learning and working environment have focused on trainee work hour limits [30, 31] and interventions to promote a safe, effective, and supportive learning environment [12].

Suggestions for broader interventions have generally been made at the conceptual level, such as proposals in the United States to reallocate funding for graduate medical education to incentivize programs that meet social accountability aims [32], and some commentators have recommended metrics to be used in the allocation of funds [33]. In Canada, the report of a consortium of organizations that addressed the future of postgraduate medical education emphasized the social accountability of physicians to the public they serve [34]. At the undergraduate medical education level, a US proposal for a “social mission” metric assessed the output of medical schools as the percentage of graduates who practice primary care, work in areas affected by a shortage of health professionals, and are members of under-represented minorities; their ranking showed that medical schools vary substantially in their contribution to these social missions [35].

Meeting goals for social responsiveness in a competency-based postgraduate medical education system requires accrediting organizations to ensure that learning emphasizes relevant competencies, and that graduates possess the attributes to meet societal goals for health care access, population health, improved patient experience of care, and stewardship of finite health care resources [8]. In the United States, accreditation standards that will become effective in 2019 call for postgraduate medical education programs to use a “deliberate design” approach to set aims relevant to the “needs of the communities they serve, within the overall mission of their sponsoring institution, and the capabilities of physicians they intend to graduate” [36]. The new standards also include an expectation that workforce diversity be considered in program evaluation and improvement [20]. Standards like these will increase the ability of postgraduate medical education to influence change that allows the physician education system to better meet societal needs.

### Overcoming challenges to meeting societal expectations

Strategies to improve the social accountability of postgraduate medical education can be impeded by time constraints, competition with other curricular components, financial limitations, and institutional resistance. The promotion of social accountability through accreditation will need to be sensitive to the fact that accredited programs and sponsoring institutions are complex adaptive systems [37] influenced by the environments in which they operate. This constrains their ability to meet the range of potentially competing demands that arise from oversight organizations and the varying interests and foci within the medical education community. For example, in the Netherlands, competing perspectives with regard to accountability, educational focus, trust, role modelling, and work-life balance emerged in discussions about the future of the nation’s postgraduate medical education system [38].

There are additional challenges with regard to efforts to optimize the size and mix of the physician workforce (Table 1) in largely market-based systems such as the United States and, to a lesser degree, Canada. Accreditation cannot be used as a direct lever, as accreditors generally are enjoined from using workforce adequacy determinations in accreditation decisions [39]. In addition, while formal curricula and learning experiences can promote generalist practice and care of underserved populations, efforts to increase the supply of physicians for generalist practice and care in these populations need to address the “hidden curriculum” [40] in medical education, including the disparagement of generalist specialties [41] and the financial [42] and societal [41] reward structure for these highly needed specialties.

Globally, there are concerns with the “brain drain” that results from the migration of physicians to nations with greater freedom, higher reimbursement, and better quality of life, or to address local physician shortages [43]. In 2011, the WHO instituted a Global Code of Practice on the International Recruitment of Health Personnel, in recognition of the fact that the out-migration of physicians may hurt nations that have a high disease burden and already face shortages in their health workforce [14]. Efforts to address physician shortages in Australia and New Zealand have suggested that aligning elements of education and health systems with patient care needs is more effective than “importing” physicians [44], and in the United Arab Emirates there are efforts to train Emirati nationals to make up the next generation of physicians to serve the nation and region [45].

Addressing societal needs and expectations in postgraduate medical education is an area for additional work, including research on effective approaches and outreach to stakeholders, exploring successes and challenges, and gathering and disseminating information on effective practices for adoption or adaptation.

## Conclusion

Societal responsiveness in postgraduate medical education can be achieved through a range of approaches at the system, institution, program, and individual levels. Accreditation has the potential to be effective across different national contexts and across the various dimensions of social responsiveness. A key observation is that meeting social responsiveness in a competency-based postgraduate medical education system requires accrediting organizations to ensure that learning emphasizes relevant competencies, and that graduates possess desired attributes. At the same time, there is a role for institutions that sponsor graduate medical education to provide safe and effective patient care in a supportive, humanistic learning and working environment.

## Data Availability

Not applicable.

## Abbreviations

WFME: World Federation for Medical Education
WHO: World Health Organization

## Footnotes

1 This discussion, held at the 2013 World Summit on Outcomes-Based Accreditation in Calgary, Alberta, in conjunction with the International Conference on Residency Education, included representation across the continuum of medical education (undergraduate, postgraduate, and continuous professional development) and from a number of nations. The participants, who represented a variety of roles in medical education and accreditation, included current and former program directors and deans, senior administrators, and accreditors.
